# Study on the Mechanical Properties and Energy Absorbing Capability of Polyurethane Microcellular Elastomers under Different Compressive Strain Rates

**DOI:** 10.3390/polym15030778

**Published:** 2023-02-03

**Authors:** Zhiying Zhao, Xiaodong Li, Hao Jiang, Xing Su, Xudong Zhang, Meishuai Zou

**Affiliations:** School of Materials Science and Engineering, Beijing Institute of Technology, Beijing 100081, China

**Keywords:** PUME, strain rate, energy absorption and efficiency diagrams, constitutive model

## Abstract

Polyurethane microcellular elastomers (PUME) are good at impact protection and energy absorption, and belong to rate sensitive- and strain history-dependent materials. In this study, PUME with different densities of 800 kg/m^3^, 600 kg/m^3^ and 400 kg/m^3^ were prepared, then the compressive responses of PUME in the strain rate range of 0.001 s^−1^ to 3400 s^−1^ were systemically investigated. By studying the energy absorption and efficiency diagram of PUME, the compressive properties of materials with different densities under compressive impact load were described, which showed that PUME with a density of 600 kg/m^3^ had better performance. A visco–hyperelasticity–air constitutive model was established to describe the large deformation response of PUME at high strain rates. The model included three components: hyperelastic part, viscoelastic part and gas pressure part. Quasi-static and dynamic compression tests were used to determine the constitutive relations of seven parameters. The samples with a density of 600 kg/m^3^ at different strain rates were fitted by MATLAB software, and the constitutive model parameters were obtained. The comparison between the constitutive equation and the experimental results showed that there was a good consistency. The constitutive model can provide data support for simulation analysis and application of PUME as energy absorbing protective facilities.

## 1. Introduction

Excellent energy absorbing materials absorb kinetic energy, mainly through plastic collapse, and they bear high impact loads and show high energy absorption efficiency. They are widely used in protective facilities with special fields, such as collision, explosion, ballistic impact, etc. The materials with multi-cell structure have the advantages of low density, high specific strength and good energy absorption. Multicellular materials include lattice materials and foam materials.

Compared with epoxy resin and polymethylmethacrylate, polyurethane elastomers (PUE) have higher load-bearing, cutting resistance, wear resistance, tear resistance, sound absorption and energy absorption capabilities, so they are often regarded as a multi-functional material [1,2,3,4,5]. PUE are a copolymer composed of a hard segment (a diisocyanate and chain extender) and a soft segment (a polyether or polyester polyol). The –NH, as the electron donor group, can form hydrogen bonding with the electron-absorbing carbonyl oxygen (C=O) in the hard segment, which is conducive to the formation of a physical cross-linking network and microphase separation structure. Therefore, PUEs can adapt to different mechanical conditions by changing their microstructure [6,7,8].

Polyurethane microcellular elastomer (PUME) has the same properties as both foam and elastomer materials, so it can be used as an efficient energy absorbing material. After being subjected to severe impact loading, foam materials absorb energy through cell wall buckling and collapse damage [9] and energy stored by air compression. Elastomer materials convert mechanical energy into internal energy through polymer chain unwinding, fracture and crack propagation [10]. At present, researchers have studied the energy absorption of rigid polyurethane foam and expanded polypropylene. Linul et al. [11] conducted dynamic compression tests on seven kinds of rigid polyurethane foams with different densities at the same impact loading speed. According to the measured loading displacement curve, the peak stress, energy absorption and efficiency properties of each foam were investigated, and the best foam density was selected. Avalle et al. [12] studied the quasi-static compression response of three polymer foams (namely expanded polypropylene, rigid polyurethane foam and PS/PA foams) at room temperature. The energy absorption characteristics were studied by energy absorption diagram and efficiency diagram. Santos Da Campo et al. [13] studied the correlation between electrostatic potential and fatigue failure in elastomers during the stretching process, and they found the convenient features of electromechanical coupling in rubbers for non-contact and real-time prediction of fatigue failure. However, there is no relevant research report on the energy absorption of PUME under high-speed impact conditions, which restricts its application in the field of impact energy absorption.

The constitutive relationship of PUME is an important element of its mechanical research. It is also a key problem in mechanical analysis and numerical simulation, which directly affects its application in the field of energy absorption [14,15,16]. In recent years, some constitutive relations of rigid polyurethane foams and rubber elastomer materials under quasi-static and dynamic loads have been established through the unremitting efforts of many scholars. Yang et al. [17] studied the dynamic and quasi-static compression response of two polyurethane foams with different densities, and they established a constitutive model, proving that it was a strain rate sensitive material. The proposed model could describe the viscoelastic hyperelastic behavior of compressible elastomer materials loaded at high strain rates. Fan et al. [18] prepared polymer composites by blending methyl methacrylate and soft dioctyl phthalate. Compressive tests were carried out under quasi-static and dynamic loads at room temperature. The yield stress increased with the increase in strain rate, which was rate dependent and described by a quantitative function.

However, due to the complexity of dynamic and quasi-static mechanical properties of various PUMEs, as well as the lack of dynamic and quasi-static experimental data, the research on the dynamic and quasi-static constitutive model of PUME was very limited. In addition, during the forming process of PUMEs, there was chemical foaming reaction, and the foam cells were filled by gas. The gas inside the cells will have a certain impact on the mechanical behavior of the materials. Therefore, it is necessary to establish a more suitable constitutive model to describe the mechanical properties of PUME on the basis of the existing nonlinear viscoelastic theory of elastomers, including the contribution of intracellular gas.

This work systematically studied the mechanical properties of a new type of PUME under dynamic and quasi-static load at room temperature, which revealed the strain rate and density dependence of mechanical properties. The most suitable foam density was obtained by studying the energy absorption and absorption efficiency of PUME with different densities. The constitutive model of PUME was established, and the parameters of the constitutive equation were determined by MATLAB software. The constitutive equation was then compared with the experimental results. The effective establishment of the constitutive equation has a guiding significance for the design of the cushioning energy absorption structure of the materials used in packaging protection engineering.

## 2. Materials and Methods

### 2.1. Materials

Polytetrahydrofuran ether glycol (PTMEG, technical pure), with the molecular weight of 1000 and 2000, and 4,4′-diphenylmethane diisocyanate (MDI, technical pure) were purchased from Wanhua Chemical Group Co., Ltd., Yantai, China. Poly (propylene oxide) triol (EP330, technical pure), with the molecular weight of 5000, was supplied by Shandong Bluestar Dongda Co., Ltd., Zibo, China. 1,4-Butanediol (BDO), analytical purity, was purchased from Shanghai McLean Biochemical Technology Co., Ltd., Shanghai, China. Bis (dimethylaminoethyl) ether (BDMAEE), which was analytical pure, was derived from Shanghai Ye Innovation Materials Co., Ltd., Shanghai, China. Catalyst dibutyltin dilaurate (T12, analytical pure) was purchased from Shanghai Xindian Chemical Materials Co., Ltd., Shanghai, China. Foam homogenizing agent (AK7703, analytical pure) was provided by Jiangsu Maysta Chemical Co., Ltd., Nanjing, China. Deionized water, which was self-made, acted as foaming agent.

### 2.2. Preparation of PUME

#### 2.2.1. Dehydration of Raw Materials

For the PUME samples prepared in this study, water acted as foaming agent and chain extender. As the hydroxyl value of water is high, it was necessary to control the water content of the raw materials in this experiment. Accordingly, the raw materials, including EP330, PTMEG1000, PTMEG2000 and BDO, were pretreated by dehydration. The dehydration conditions were as follows: temperature was set from 105 °C to 110 °C; vacuum degree was below 0.01 MPa; dehydration time was 2 h. The dehydration requirements could be met when the water residual content in the samples when measured was between 1/10,000 and 2/10,000 by the automatic micro moisture analyzer.

#### 2.2.2. Preparation of PUME

Preparation of component A: mixing specific amount of PTMEG, chain extender (BDO), catalyst (T12), AK7703 and H_2_O.

Preparation of component B: adding metered polyether polyol and MDI to the three-port flask equipped with thermometer and agitator. The temperature was raised to about 85~90 °C in N_2_ protective atmosphere, and the reaction was sustained for 4 h under constant stirring.

**Figure 1:**
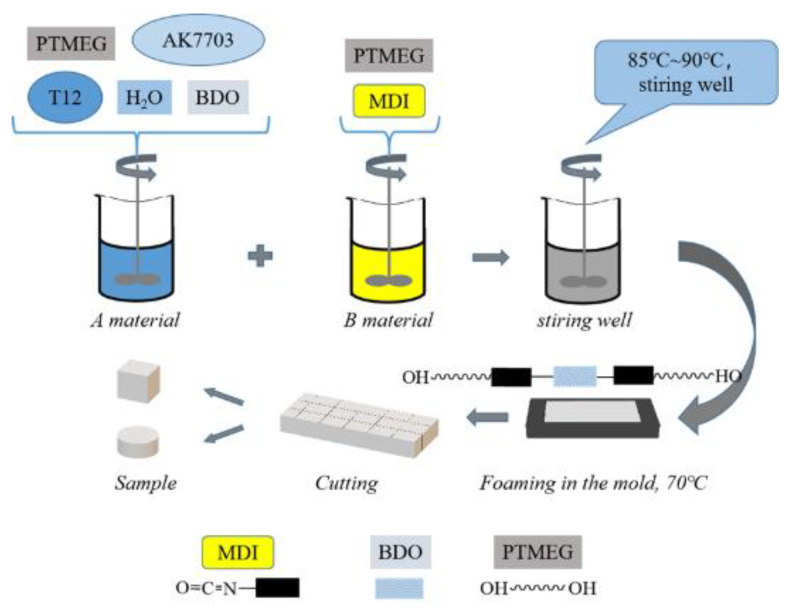
Technological process of preparing PUMEs.

The A and B components were mixed in a certain proportion (R = 1.0), then the foam was removed in vacuum and poured into the plate mold coated with release agent. The PUME was obtained by placing it in a blast drying oven at 70 °C for 24 h, followed by another 7 days storage at a temperature of 23 °C ± 2 °C and humidity of 50% ± 5%. The synthetic process is illustrated in Figure 1. PUMEs with different densities could be prepared by changing the amount of raw materials. The density range of PUME is about 300 kg/m^3^~1200 kg/m^3^. We chose the representative low, medium and high densities, which were 400 kg/m^3^, 600 kg/m^3^ and 800 kg/m^3^. The densities of different samples were controlled at 400 kg/m^3^, 600 kg/m^3^ and 800 kg/m^3^ by fine-tuning the amount of foaming agent H_2_O and the quantity of raw materials injected into the mold. The synthetic formula of PUMEs with different densities is shown in Table 1. We then carried out comparative experimental research to obtain the best energy absorption utilization rate.

The tested samples of PUMEs were made using a cutting tool. Samples with the dimension of 100 mm × 100 mm × 100 mm were prepared for quasi-static load compression test, and Φ 16 mm × 5 mm were prepared for dynamic load compression test.

### 2.3. Measurements and Characterization

#### 2.3.1. Infrared Spectrum Analysis

Fourier transform infrared spectra (FTIR) of samples were recorded by an IRAffinity-1S spectrometer (Shimadzu company, Japan), Each sample was scanned by 10 cycles in a wavenumber range from 4000 cm^−1^ to 500 cm^−1^ at a resolution of 5 cm^−1^. The test temperature was 23 °C ± 2 °C.

#### 2.3.2. Characterization by Scanning Electron Microscopy

The morphology of the original samples was observed by scanning electron microscope (SEM) (TM3030, HITACHI, Japan). The samples were cut into pieces and then sputtered and plated for SEM observation. The acceleration voltage was 15 kV, and the magnification was 100. The test temperature was 23 °C ± 2 °C.

#### 2.3.3. Quasi-Static Mechanical Property Test

The quasi-static compression tests of PUMEs were carried out on an Instron tester (8872) at the strain rate from 0.1 s^−1^ to 0.001 s^−1^ [18]. The samples were compressed to more than 60% of the original thickness. Three samples were tested for each material, and the quasi-static compression tests were carried out at the strain rates of 0.001 s^−1^, 0.01 s^−1^ and 0.1 s^−1^, respectively. The sample size was 100 mm × 100 mm × 100 mm. The test temperature was 23 °C ± 2 °C.

#### 2.3.4. Dynamic Mechanical Property Test

**Figure 2:**
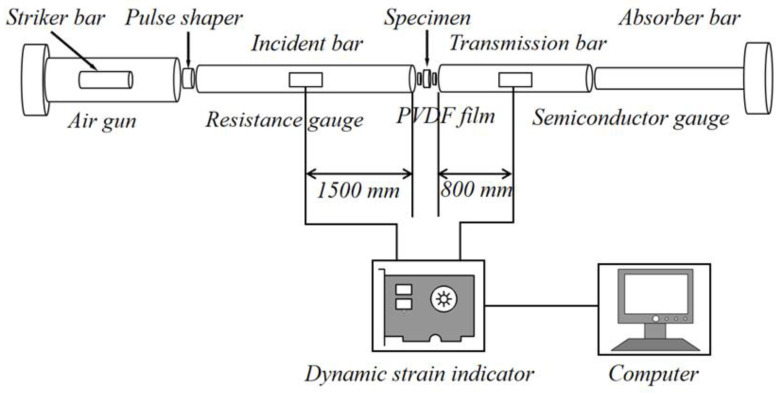
SHPB experimental device.

The compression test adopted a modified Split Hopkinson Pressure Bar (SHPB) [19,20], which is widely used for studying the dynamic properties of composite foam materials. As shown in Figure 2, the strain history recorded by strain gauges fixed on the incident rod and transmission rod was converted into a stress–strain curve [9,21,22] using the following methods.

(1) $$\dot{\varepsilon }=-\frac{2C_{0}}{l_{s}}\varepsilon_{r}$$

(2) $$\varepsilon =\int_{0}^{t}\dot{\varepsilon }\;\mathrm{dt}=\int_{0}^{t}\frac{2c_{0}}{l_{s}}\varepsilon_{r}\mathrm{dt}$$

(3) $$\sigma =\frac{A_{0}}{A_{s}}\cdot E{\;\varepsilon }_{t}$$

where l_s_ and A_s_ are the original sample length and cross-sectional area, respectively; E, C_0_ and A_0_ are Young’s modulus, elastic wave velocity and cross-sectional area of the bar, respectively; $\varepsilon_{r}$ and $\varepsilon_{t}$ are the strain signals reflected and transmitted by the rod surface, respectively.

The diameter of the impact rod, incident rod and transmission rod was 50 mm. The length of the incident rod and the transmission rod was 3000 mm, and the length of the impact rod was 1000 mm. The bar was made of aluminum, with a density and Young’s modulus of 2.7 × 10^3^ kg/m^3^ and 73 GPa, respectively. The test temperature was 23 °C ± 2 °C.

**Figure 3:**
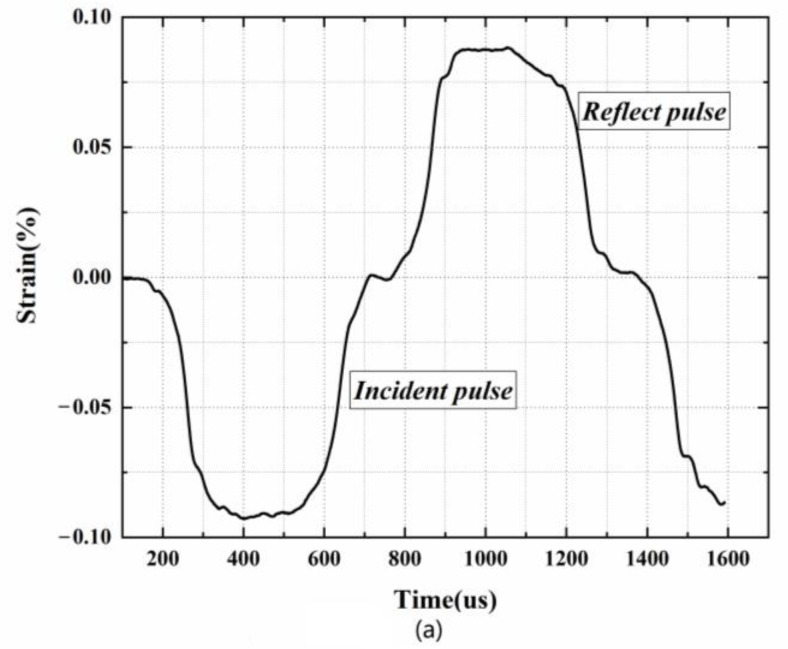

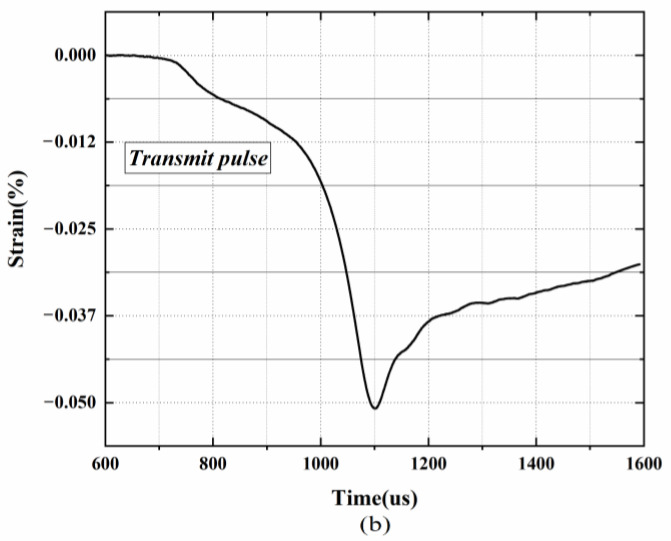
Typical PUME waveforms in SHPB experiments: (**a**) incident and reflected waves, (**b**) transmitted waves.

Figure 3 shows the oscilloscope recording of incident, reflected and transmitted signals of the PUME with a density of 400 kg/m^3^ in SHPB tests at 2500 s^−1^ strain rate. The incident, reflected and transmitted waves are clear and complete, which proves that the SHPB experimental device can well analyze the dynamic compression properties of PUMEs.

## 3. Results and Discussion

### 3.1. FTIR Results

In addition to determining the microstructure of substances, infrared spectroscopy is very sensitive to hydrogen bonding. It is widely used in studying the hydrogen bonding force and phase separation behavior in polyurethane systems [23]. Hydrogen bonding force is widespread and very important in PUMEs. It forms a physical cross-linking network, which could effectively enhance the mechanical properties of PUMEs.

**Figure 4:**
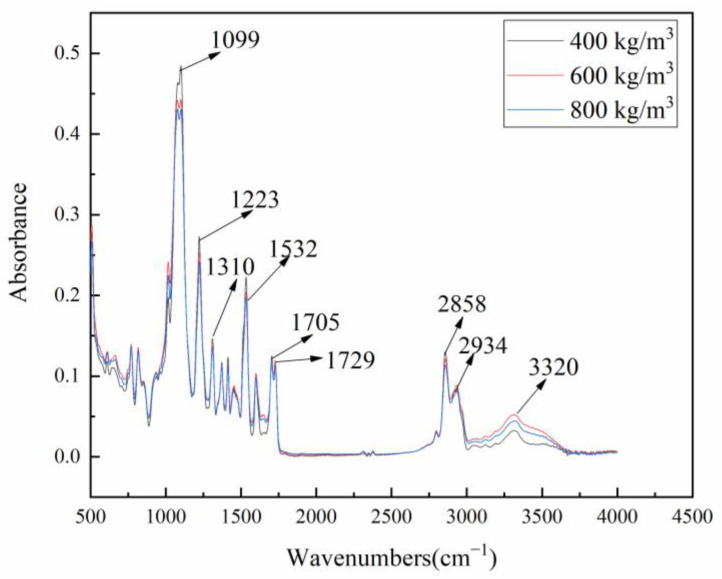
FTIR spectra of the PUMEs at 23 °C ± 2 °C.

The infrared spectrum of PUMEs is shown in Figure 4. The samples with different densities of 800 kg/m^3^, 600 kg/m^3^ and 400 kg/m^3^ were prepared by the same formula, and the infrared spectra basically coincided. It can be clearly seen that the characteristic absorption peaks of -NCO at 2270 cm^−1^ and -NH at 3480 cm^−1^ disappeared, while the characteristic absorption peaks of -NH and -CO appeared near 3320 cm^−1^ and 1730 cm^−1^, which indicates that -NCO reacted with -OH to form carbamate. PUMEs were successfully synthesized [24]. The observed frequency bands representing the peak vibrations are listed in Table 2.

The hydrogen bonds between hard segments are helpful in forming the microphase separation structure of PUME. As an electron donor group, -NH- in carbamate could form hydrogen bonding with electron-absorbing carbonyl and ether bonds [25,26]. The hydrogen bonds can be formed between the active hydrogen in -NH- and the oxygen in C=O in polyurethane polymer, and the strength of the hydrogen bonds directly affect the ordered structure of the hard segments. The infrared absorption peak of free -NH- belonged to 3445 cm^−1^~3450 cm^−1^, and they moved to 3315 cm^−1^~3340 cm^−1^ after hydrogen bonding formed [7]. The infrared spectrum of PUME shows that the hydrogen-bonded -NH- absorption peak appeared near 3320 cm^−1^, indicating that a microphase separation structure was formed in PUME.

**Figure 5:**
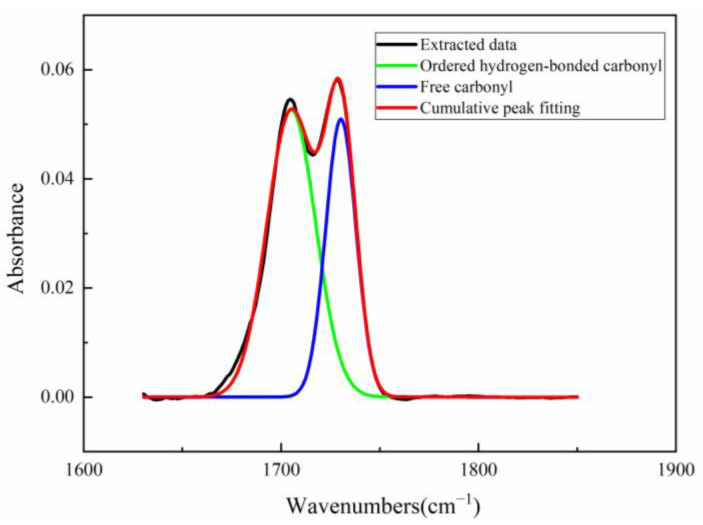
FTIR Spectra of carbonyl region for PUME at 23 °C ± 2 °C.

In order to quantitatively study the degree of hydrogen bonding, the FTIR spectra of the carbonyl region were analyzed. As shown in Figure 5, multiple bands of PUME in 1660 cm^−1^~1760 cm^−1^ could be observed. After deconvoluting the carbonyl region in the figure, different peaks could be obtained, corresponding to the different states of urethane carbonyl forming hydrogen bonds, in which 1690 cm^−1^~1709 cm^−1^ are ordered carbonyl hydrogen bonds, 1710 cm^−1^~1724 cm^−1^ are disordered carbonyl hydrogen bonds and 1725 cm^−1^~1740 cm^−1^ are free carbonyl groups [25].

The least square method was used to fit the above carbonyl zone curve and divide the peaks [26]. The results of peak separation are listed in Table 3. The ordered hydrogen bonding degree (X_O,UA_), the disordered hydrogen bonding degree (X_D,UA_) and the total hydrogen bonding degree (X_B,UA_) of carbonyl are defined as:

(4) $$X_{O,\mathrm{UA}}=\frac{\sum^{\;}\mathrm{Area}\left(\mathrm{ordered}\right)}{\sum^{\;}\left[\mathrm{Area}\left(\mathrm{ordered}\right)+\mathrm{Area}\left(\mathrm{disordered}\right)+\mathrm{Area}\left(\mathrm{free}\right)\right]}$$

(5) $$X_{D,\mathrm{UA}}=\frac{\sum^{\;}\mathrm{Area}\left(\mathrm{disordered}\right)}{\sum^{\;}[\mathrm{Area}\left(\mathrm{ordered}\right)+\mathrm{Area}(\mathrm{disordered})+\mathrm{Area}\left(\mathrm{free}\right)]}$$

(6) $$X_{B,\mathrm{UA}}=X_{O,\mathrm{UA}}+X_{D,\mathrm{UA}}$$

where Area(ordered), Area(disordered), Area(free) represent the peak area of ordered hydrogen bonding, disordered hydrogen bonding and free carbonyl, respectively.

It can be seen that the hydrogen bonding degree in PUME is high, which may cause it to aggregate or polymerize into ordered hard segments, while the soft segments form amorphous domains, resulting in micro phase separation. This structural feature gives PUMEs higher modulus and elasticity.

### 3.2. SEM Results

**Figure 6:**
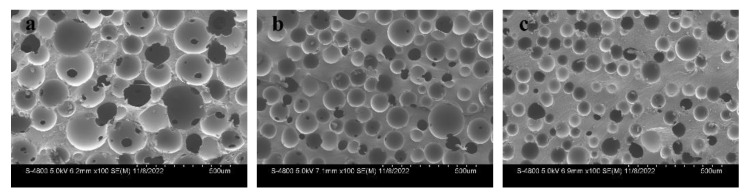
SEM microstructures of the PUMEs with densities of (**a**) 400 kg/m^3^, (**b**) 600 kg/m^3^ and (**c**) 800 kg/m^3^ at 23 °C ± 2 °C.

**Figure 7:**
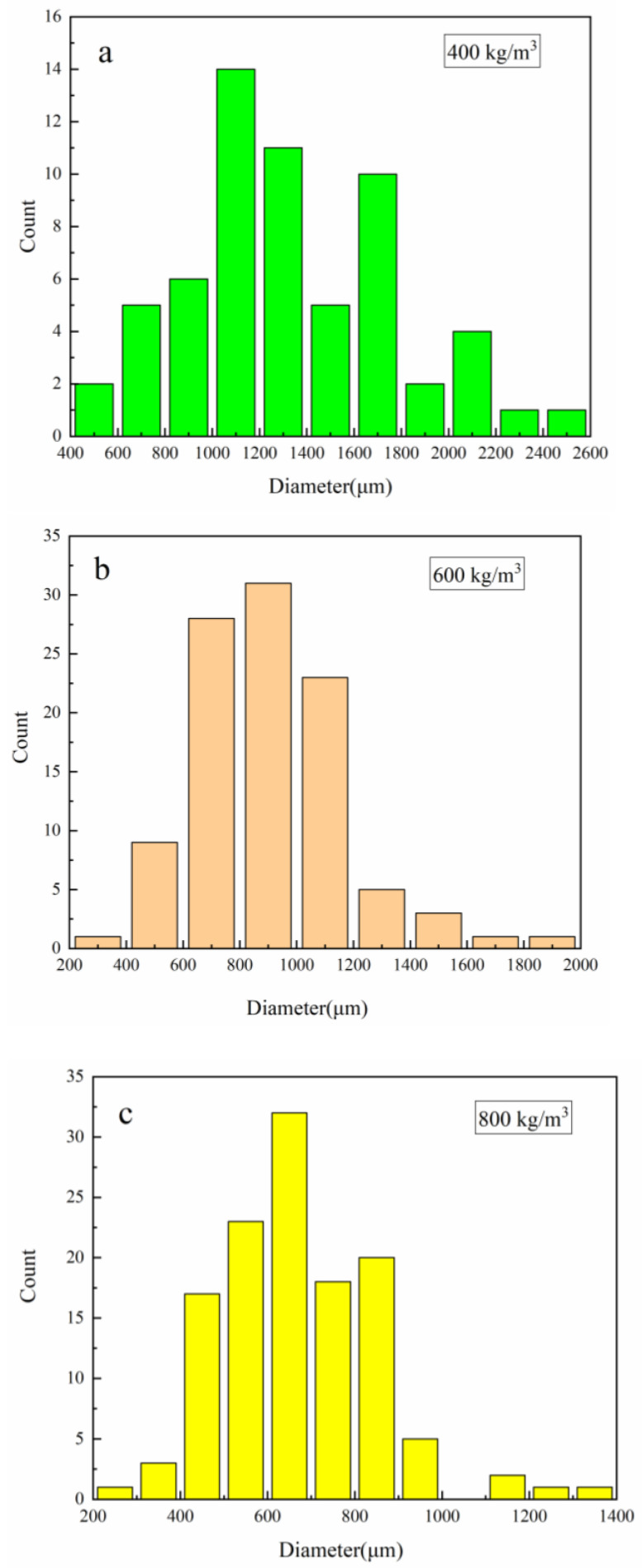
The distribution of the cell diameters: (**a**) 400 kg/m^3^, (**b**) 600 kg/m^3^ and (**c**) 800 kg/m^3^.

PUMEs with different densities were observed by SEM, as shown in Figure 6. The micrographs were imported into the ImageJ analysis software to obtain the sizes of the cells in the pictures. The distribution of the cell diameters are shown in Figure 7. It was found that the larger the sample density was, the larger the pore gap and matrix skeleton were, and the cell diameter became smaller.

### 3.3. Quasi-Static and Dynamic Compression

**Figure 8:**
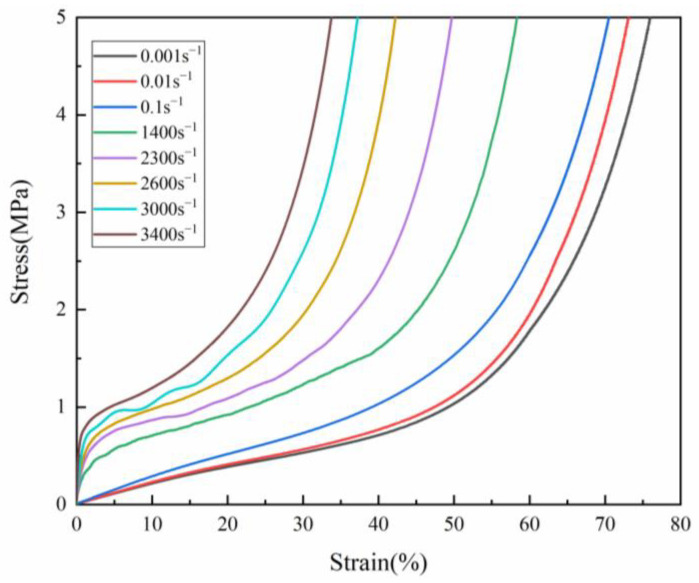
The stress–strain curves of 600 kg/m^3^ PUMEs with different strain rates at 23 °C ± 2 °C.

In order to study the mechanical response of PUMEs, compression tests with different strain rates were carried out. Figure 8 shows the stress–strain curves of the 600 kg/m^3^ PUME at different high strain rates, which are 0.1 s^−1^, 0.01 s^−1^, 0.001 s^−1^, 1400 s^−1^, 2300 s^−1^, 2600 s^−1^, 3000 s^−1^ and 3400 s^−1^. It can be seen that PUMEs belonged to strain rate-sensitive materials, and the stress increased obviously with increasing strain. For the curve of low strain rate compression, the interface between the elastic zone and the platform was not obvious, and there was almost no platform stage. It jumped directly from the elastic stage of low elastic modulus to the elastic stage of high elastic modulus. This might be because the stress in quasi-static compression had enough time to transfer to the cells and molecular chains, and there was sufficient time for cell deformation and molecular chain movement. The dynamic compression stress–strain curves at high strain rate showed obvious elastic stage, platform stage and densification stage [18]. It may be that the molecular chains did not have enough time to move, and the deformation of the cells played a leading role. The deformation mechanism of cell collapse is that the stress concentration is first generated at the weak hole wall, forming a deformation zone; the deformation zone develops continuously and collapses layer by layer. The irregular micro deformation is the result of the joint action of the bending and folding of the hole walls. PUME is prone to deformation when facing external impacts and can maintain the stress at a low level while deforming. It consumes a significant amount of work in the process of compression deformation, and converts it into energy dissipated by plastic deformation, collapse, fracture and other forms in the structure, so as to effectively absorb external impact energy.

**Figure 9:**
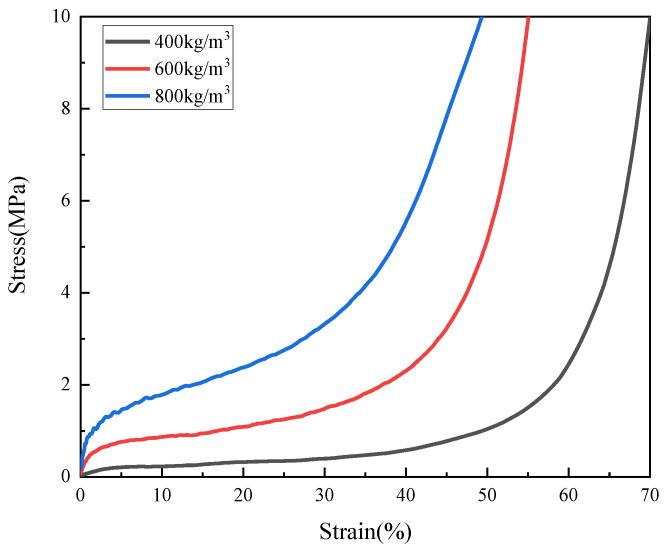
The stress–strain curves of PUMEs with different densities at a strain rate of 2300 s^−1^ and test temperature of 23 °C ± 2 °C.

In order to study the mechanical response of PUMEs with different densities, dynamic compression tests were carried out with samples of 800 kg/m^3^, 600 kg/m^3^ and 400 kg/m^3^ at the strain rate of 2300 s^−1^. The stress–strain curves are shown in Figure 9. Each density material had an obvious elastic stage, platform stage and densification stage. The stress characteristic value increased with increasing density. Combined with the results of SEM, the larger the cell gap and matrix skeleton was, the stronger the bearing capacity was, which led to the increase in the stress–strain curve with the increase in density.

### 3.4. Analytical Model for Determining Foam Energy Absorption Diagram

The purpose of the energy-absorbing materials used in packaging protection engineering is to dissipate the kinetic energy in the impact process while keeping the impact force (or acceleration) below a certain limit (critical value). For each applicable condition, the PUME with the most suitable density could be found. If the density was too low, it reached the dense area before all the energy dissipated, and the impact force (or acceleration) was still large. If the density was too high, the force exceeded the critical value before absorbing enough energy, and the compressive strain of the material was only partially utilized.

The energy absorption capacity of materials can be characterized by the definition of energy absorption per unit volume [11,12], as shown in Equation (7). The samples with densities of 800 kg/m^3^, 600 kg/m^3^ and 400 kg/m^3^ were selected under the loading rate of 11.5 m/s.

(7) $$W=\int_{0}^{\varepsilon }\sigma \left(\varepsilon \right)d\varepsilon$$

**Figure 10:**
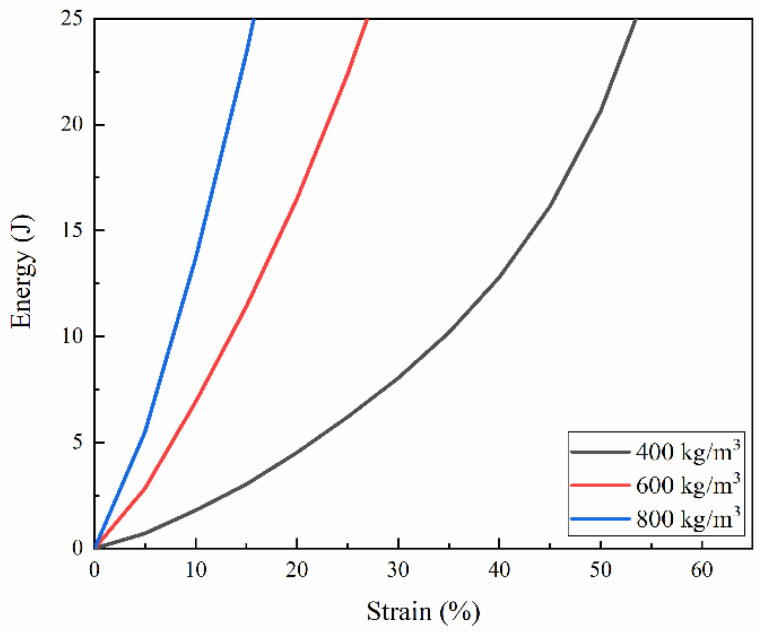
Energy–strain curves of PUMEs resulted from dynamic compression tests at 23 °C ± 2 °C.

**Figure 11:**
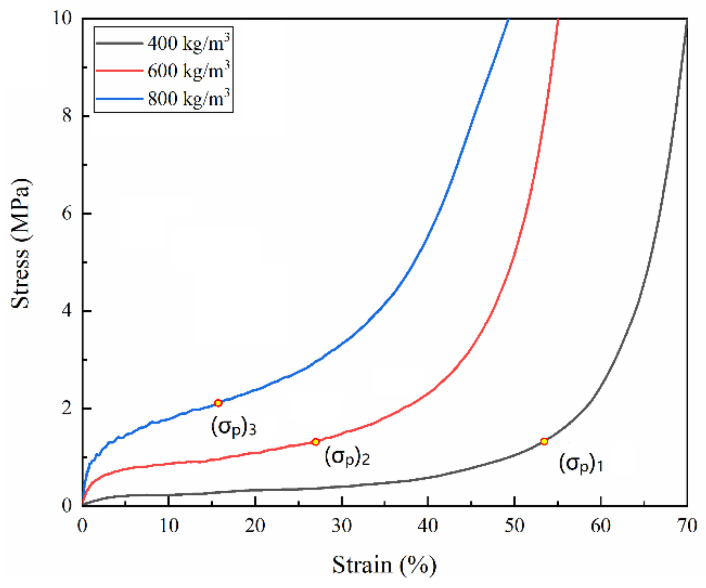
The peak stress generated in different foams when absorbing the same amount of energy at 23 °C ± 2 °C.

**Figure 12:**
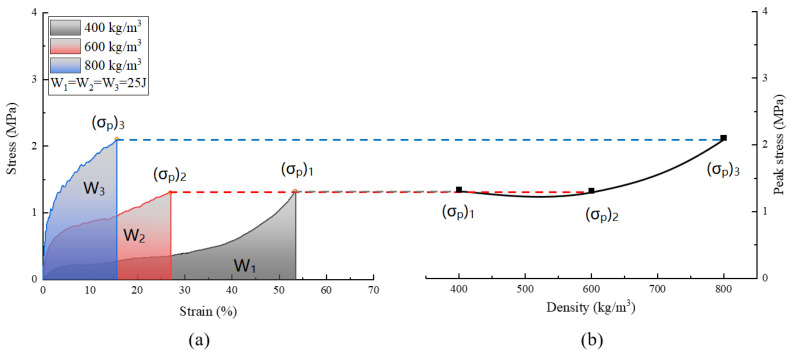
Energy-absorption diagram: (**a**) detailed stress–strain curves; (**b**) peak stress variation depending on the density.

**Figure 13:**
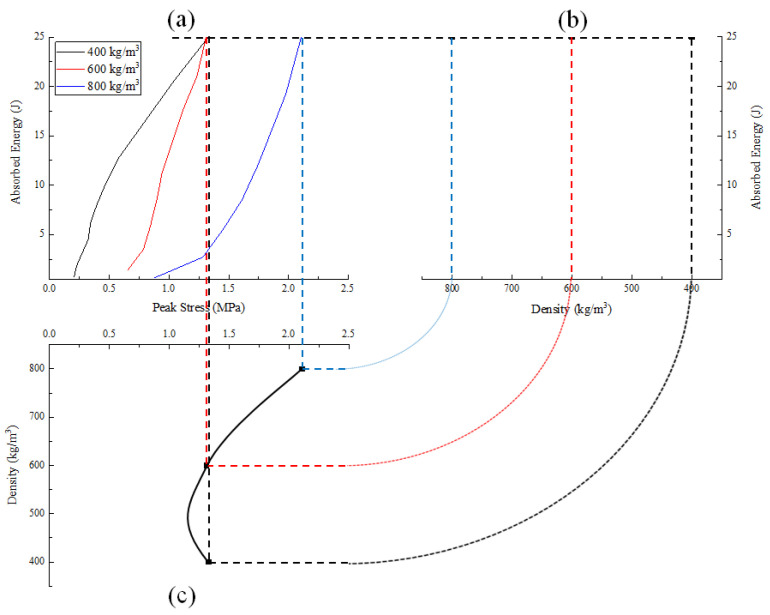
Energy absorption diagrams resulting from dynamic compression tests: (**a**) absorbed energy–peak stress curve; (**b**) absorbed energy–density curve; (**c**) density–peak stress curve.

The same energy value of 25 J for all densities was chosen, as shown in Figure 10, corresponded to the points $(\sigma_{p}){}_{1},(\sigma_{p}){}_{2}$ and $(\sigma_{p}){}_{3}$ in the stress–strain curves, as shown in Figure 11. These points were named peak stresses. Figure 12 and Figure 13 illustrate the variation of these peak stresses with density and the most suitable density of PUMEs. When absorbing energy, W_1_, the PUME with the lowest density of 400 kg/m^3^ would produce the peak stress value $(\sigma_{p}){}_{1}$ of 1.338 MPa. The PUME with the density of 800 kg/m^3^ would produce a high peak stress $(\sigma_{p}){}_{3}$ of 2.085 MPa before absorbing energy, W_3_. It can be observed that the density of 600 kg/m^3^ absorbed the same energy, W_2_, and produced a lower peak stress value $(\sigma_{p}){}_{2}$ of 1.304 MPa.

Another parameter for characterizing energy absorption is energy absorption efficiency, which is defined as the ratio of absorbed energy to stress, σ, as shown in Equation (8) [11].

(8) $$E_{\mathrm{ff}}=\frac{\int_{0}^{\varepsilon }\sigma \left(\varepsilon \right)d\varepsilon }{\sigma_{p}}$$

**Figure 14:**
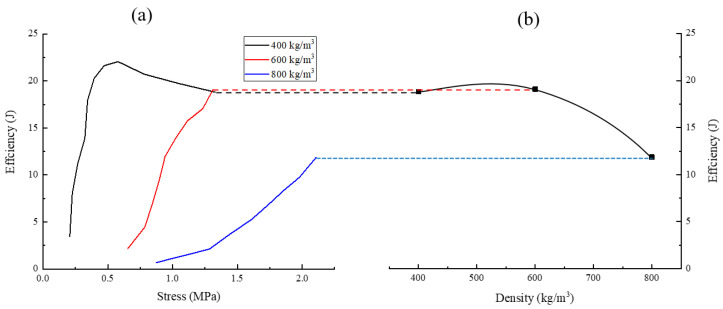
Energy-absorption diagram: (**a**) energy absorption efficiency–stress curve; (**b**) energy absorption efficiency–density curve.

The energy absorption efficiency–stress curve and energy absorption efficiency–density curve of samples are illustrated in Figure 14, which shows that the results of the energy absorption diagram and the energy absorption efficiency diagram were consistent. It can be concluded that the density corresponding to the highest energy absorption efficiency was about 510 kg/m^3^, while the PUME energy absorption efficiency with the density of 600 kg/m^3^ was greater than that, with a density of 400 kg/m^3^ and 800 kg/m^3^.

### 3.5. Constitutive Model of PUME

The relationship between the micro-phase separation structure with soft and hard segments of polyurethane and the mechanical deformation is complex. The soft segments in PUMEs can be regarded as elastic “springs” under environmental conditions, while the hard segments can be regarded as relatively short “rigid units”, which decides their microstructure and properties [7].

**Figure 15:**
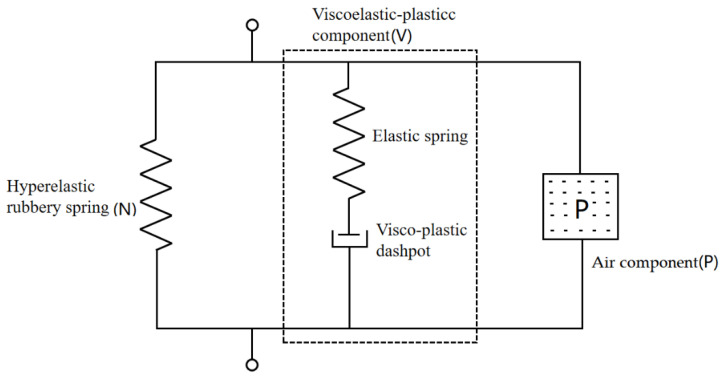
One-dimensional diagram of constitutive model.

The large strain nonlinear stress–strain behaviors of polyurethane elastomer show strong hysteresis, rate dependence and softening. The constitutive model for the two-phase structure was proposed by Mossi Idrissa [27]. In this study, a gas unit was added on the basis of the two-phase structure. The model not only considered the hyperelastic behavior of the soft segments and the elastic-plastic characteristics of the hard segments, but also the air pressure bearing characteristics in the cells. An effective constitutive model that can reasonably reflect the microstructure of PUME can be developed through its mechanical behavior. In order to rationalize the viscoelastic behavior of elastomers, the stress response of PUME can be divided into three parts: (1) hyperelastic part, (2) viscoelastic part [28] and (3) gas pressure part, as shown in Figure 15.

The compressible hyper elastic component (N) acted like a linear spring, which was used to capture the entropy change caused by the molecular orientation of the soft domain and was responsible for the high elasticity of the overall deformation. The viscoelastic-plastic component (V) consisted of a linear elastic spring and a nonlinear viscoplastic pot, which characterized the initial elastic contribution due to the change of internal energy. The nonlinear viscoplastic pot captured the rate and temperature-dependent behavior of the material. The air component (P) described the contribution of air compression to material mechanics under pressure in a closed cell structure.

In this model, it was assumed that the strain energy potential could be expressed by a newly proposed polynomial series with three independent parameters. The strain rate sensitivity was characterized by the combination of a nonlinear Maxwell relaxation model with four parameters. Dynamic and quasi-static compression response can be described by compression hyperelastic component (N), visco–hyperelasticity component (V) and air component (P).

#### 3.5.1. Strain Energy Density Function

In order to establish the visco–hyperelasticity–air constitutive model of PUME, the parameters of the model were defined, as listed in Table 4.

Assuming that the point originally located at a certain position, X, in the material is displaced to the new position, x, after deformation, the deformation gradient, F, is defined as:

(9) $$F=\frac{\partial x}{\partial X}$$

The deformation of the material can be described by the left Cauchy–Green deformation tensor B = F·F^T^, then the right Cauchy–Green deformation tensor can be described by C = F^T^·F. Green’s strain tensor E = (C − I)/2. The three invariants I_1_, I_2_ and I_3_ of B are defined as:

I_1_ = tr(B) (10)

(11) $$I_{2}\;=\;[I_{1}^{2}\;-\;\mathrm{tr}(B^{2})]/2$$

I_3_ = det(B) (12)

The tensile ratios of the three main directions are λ_1_, λ_2_ and λ_3_, respectively. In this case, the deformation tensor, B, is the same as the loading direction, and the deformation gradient, F, can be defined as:

(13) $$F=\left(\begin{array}{ccc} \lambda_{1} & 0 & 0\\ 0 & \lambda_{2} & 0\\ 0 & 0 & \lambda_{3} \end{array}\right)$$

(14) $$B=F\;F^{T}=C=F^{T}F=\left(\begin{array}{ccc} \lambda_{1}^{2} & 0 & 0\\ 0 & \lambda_{2}^{2} & 0\\ 0 & 0 & \lambda_{3}^{2} \end{array}\right)$$

J = detF and J is the volume ratio before and after deformation. It is generally considered that the hyperelastic material is incompressible. Therefore, PUME belongs to elastomer foam, and the volume is nearly constant during compression. For the volume invariant material, J = λ_1_ λ_2_ λ_3_ = 1. Under uniaxial loading, the elongation ratio in the loading direction is λ. Because the material is incompressible, the three-dimensional principal elongation ratios are λ_1_ = λ, λ_2_ = λ_3_ = λ^−1/2^.

(15) $$I_{1}\;=\;\mathrm{tr}(B)\;=\;\lambda_{1}^{2}\;+\;\lambda_{2}^{2}\;+\;\lambda_{3}^{2}\;=\;\lambda^{2}\;+\;2\lambda^{-1}$$

(16) $$I_{2}\;=\;[I_{1}^{2}\;-\;\mathrm{tr}(B^{2})]/2={\left(\lambda_{1}\lambda_{2}\right)}^{2}\;+\;{\left(\lambda_{2}\lambda_{3}\right)}^{2}\;+\;{\left(\lambda_{1}\lambda_{3}\right)}^{2}\;=\;2\lambda \;+\;\lambda^{-2}$$

I_3_ = det(B) = (λ_1_λ_2_λ_3_)^2^ = λ^2^·λ^−1^·λ^−1^ = 1 (17)

λ = 1 − ε(−ε represents compression test)(18)

I_1_ = (1 − ε)^2^ + 2(1 − ε)^−1^ (19)

I_2_ = 2(1 − ε) + (1 − ε)^−2^ (20)

(21) $$\sigma_{1}=\sigma =\frac{\sigma_{t}}{\lambda }$$

σ_2_ = σ_3_ = 0 (22)

σ_t2_ = σ_t3_ = 0 (23)

According to Rivlin’s analysis [15], the constitutive relation of isotropic compressible hyperelastic materials can be expressed as Equation (24).

(24) $$\sigma =p_{e}I+\alpha_{1}B+\alpha_{2}B\cdot B$$

The stress expression under uniaxial load is as follows:

(25) $$\sigma_{11}^{e}=-p_{e}I+\alpha_{1}B_{11}+\alpha_{2}B_{11}^{2}$$

where $\sigma_{11}^{e}$ is the Cauchy (true) stress. The relationship between the engineering stress, ${\;\sigma }_{11}^{0}$, and the real stress is $\sigma_{11}^{e}={\lambda \sigma }_{11}^{0}$. Hydrostatic pressure, $p_{e}$, is obtained from the conditions $\sigma_{22}^{e}=\sigma_{33}^{e}$=0 and $B_{22}=B_{11}^{-1/2}$.

(26) $$\sigma_{11}^{e}=\alpha_{1}\left(B_{11}-B_{11}^{-1/2}\right)+\alpha_{2}(B_{11}^{2}-B_{11}^{-1})$$

(27) $$\alpha_{1}=2I_{3}^{-1/2}(\frac{\partial W}{\partial I_{1}\;}+I_{1}\frac{\partial W}{\partial I_{2}\;})$$

(28) $${\;\alpha }_{2}=-2I_{3}^{-1/2}\frac{\partial W}{\partial I_{2}\;}$$

where σ_e_ is the Cauchy stress tensor. W = W (I_1_, I_2_, I_3_) and W is the strain energy potential. This could be expressed by polynomial series, which includes (I_1_−3), (I_2_−3) and I_3_. Among them, I_3_ characterizes the effect of volume deformation on behavior. For incompressible materials, I_3_ = 1; σ, ε and σ_t_ are nominal stress, nominal strain and real stress, respectively. For pure uniform strain, the energy density function between stress and strain is derived as follows:

(29) $$\frac{t_{1}-t_{2}}{\lambda_{1}^{2}-\lambda_{2}^{2}}=2(\frac{\partial W}{\partial I_{1}}+\lambda_{3}^{2}\frac{\partial W}{\partial I_{2}})$$

By substituting Equations (15)–(28) into (29), the constitutive relation between the hyper elastic material and the strain energy density function, W, can be obtained in Equation (30).

(30) $$\sigma_{11}^{e}=2[(1-\varepsilon )-(1-\varepsilon {)}^{-2}][\frac{\partial W}{\partial I_{1}}+{\left(1-\varepsilon \right)}^{-1}\frac{\partial W}{\partial I_{2}}]$$

Equation (30) shows that the constitutive model, which is suitable for hyperelastic materials, can be obtained by matching different strain energy density functions, which can effectively predict the mechanical behaviors of hyperelastic materials. 

#### 3.5.2. Establishment of Hyperelastic Constitutive Model

At present, researchers have constructed two kinds of classical constitutive models of hyperelastic materials based on continuum mechanical theory and thermodynamic statistics theory. Among them, the most classical continuum theory is the generalized Mooney–Rivlin model [16], as shown in Equation (31).

(31) $$W=\overset{N}{\underset{i+j=1}{\sum }}C_{\mathrm{ij}}{\left(I_{1}-3\right)}^{i}{\left(I_{2}-3\right)}^{j}+\overset{N}{\underset{i=1}{\sum }}\frac{1}{D_{i}}{\left(J-1\right)}^{2i}$$

where C_ij_ is the material constant and D_i_ is the material parameter, which indicate the compressibility of the material. According to Equation (17), for incompressible materials, J = 1. However, N values greater than 2 are rarely used when considering the first and second invariants.

The Tree–Item model is adopted, which is simplified by the generalized Mooney–Rivlin model.

(32) $$W=C_{10}(I_{1}-3)+C_{01}(I_{2}-3)+C_{20}{\left(I_{2}-3\right)}^{2}$$

#### 3.5.3. Establishment of Viscoelastic Constitutive Model

The stress of viscoelastic materials depends on the history of strain and strain rate. The constitutive relation of homogeneous and isotropic materials could be expressed in the following form [15].

(33) $$\sigma^{v}=-{\;p}^{v}I+F\left(t\right)\cdot \underset{\tau =-\infty }{\dot{\Omega \;}}\left\{C\left(\tau \right)\right\}\cdot F^{T}\left(t\right)$$

m(t) is a relaxation function, and m is expressed in the form of Prony series. The relaxation function m(t) decreases with t. Generally, m(t) can be modeled by a series of relaxation responses of parallel Maxwell elements:

(34) $$\;\;\;m\left(t-\tau \right)=\overset{N}{\underset{i=1}{\sum }}\exp\left(-\frac{t-\tau }{\theta_{i}}\right)\;$$

In the equation, $\sigma_{v}$ is the Cauchy stress tensor, $p^{v}$ is the pressure of the viscoelastic material, Ω is a matrix functional, which describes the effect of strain history on stress and is independent of the coordinate system. It could be defined as follows:

(35) $$\underset{\tau =-\infty }{\dot{\Omega \;}}=\int_{-\infty }^{t}m\left(t-\tau \right)\dot{E}\left(\tau \right)d\tau$$

Similarly, in the compression process, the deformation perpendicular to the loading direction is negligible, and the strain rate in the compression direction is assumed to have no effect on the response perpendicular to that direction. The functional Ω of finite deformation could be defined by the following equation:

(36) $$\underset{\tau =-\infty }{\dot{\Omega \;}}\left\{C\left(\tau \right)\right\}=\int_{-\infty }^{t}\phi \left(I_{1}^{\prime }\;,I_{2}^{\prime },I_{3}^{\prime }\right)\cdot m\left(t-\tau \right)\dot{E}\left(\tau \right)d\tau$$

The three invariants of C(τ) are the $I_{i}^{\prime }$ (i = 1, 2, 3). $\dot{E}$ is the strain rate.

(37) $$\dot{E}=\frac{1}{2}(\dot{F^{T}}\cdot F+F^{T}\dot{F})$$

where $\theta_{i}$ is the relaxation time and N is the order of the Prony series. With the increase in N value, the model is closer to the real material properties. Generally, a good material model does not need too many parameters to describe the basic characteristics of its behavior. Therefore, in this study, only one relaxation time, N = 1, was used.

In Equation (38), the strain energy function, Φ, is guided by the purpose of minimizing the number of parameters and the shape of the experimental dynamic stress–strain curve of polyurethane elastomers.

(38) $$\phi \left(I_{1}^{\prime }\;,I_{2}^{\prime },I_{3}^{\prime }\right)=B_{10}+B_{20}\left(I_{2}^{\prime }-3\right)+B_{30}{\left(I_{2}^{\prime }-3\right)}^{2}$$

$I_{2}^{\prime }$ is the second invariant of C (τ), $I_{2}^{\prime }$= $I_{2}$. The starting point of time is the beginning of loading, and it is assumed that the influence of the deformation history of τ<0 on the stress of time t > 0 is ignored. Therefore, the influence of pre-loading deformation history on stress is ignored.

(39) $$\underset{\tau =-\infty }{\dot{\Omega \;}}\left\{C\left(\tau \right)\right\}=\int_{0}^{t}\left\{B_{10}+B_{20}\left(I_{2}^{\prime }-3\right)+B_{30}{\left(I_{2}^{\prime }-3\right)}^{2}\;\right\}\cdot m\left(t-\tau \right)\dot{E}\left(\tau \right)d\tau$$

By bringing Equation (39) into Equation (33), we can obtain the finite strain viscoelastic model of PUMEs.

(40) $$\sigma^{v}=-{\;p}^{v}I+F\left(t\right)\cdot \int_{0}^{t}\left\{B_{10}+B_{20}\left(I_{2}^{\prime }-3\right)+B_{30}{\left(I_{2}^{\prime }-3\right)}^{2}\right\}\cdot m\left(t-\tau \right)\dot{E}\left(\tau \right){d\tau F}^{T}\left(t\right)$$

#### 3.5.4. Establishment of Air Model Establishment

During compression, the influence of gas pressure inside the cells of PUMEs could be defined by Equation (41). $V_{0}$ and $\rho^{*}/\rho^{s}$ are material volume and relative density, respectively. The volume is reduced from $V_{0}$ to V.

(41) $$\frac{V}{V_{0}}=1-\varepsilon \left(1-2\nu \right)$$

where ν is the Poisson’s ratio of the foam material, which was taken as 0.2; $\rho^{*}$ is the density of the foam material, which was taken as 600 kg/m^3^; $\rho^{s}$ is the density of the solid material used to manufacture the foam material, taken as 1150 kg/m^3^.

When the PUMEs were pressed, the inner cells of the material deformed, and the gas volume changed from $V_{g}^{0}$ to $V_{g}$.

(42) $$\frac{V_{g}}{V_{g}^{0}}=\frac{1-\varepsilon \left(1-2\nu \right)-\rho^{*}/\rho^{s}}{1-\rho^{*}/\rho^{s}}$$

The initial pressure, $P_{0}$, increases to P after compression in the cells, and $P_{0}$ is usually atmospheric pressure.

(43) $${\mathrm{PV}}_{g}=P_{0}V_{g}^{0}$$

The air pressure in the cells to be overcome during compression is as shown.

(44) $$\Delta P=P-P_{0}=\frac{P_{0}\varepsilon \left(1-2\nu \right)}{1-\varepsilon \left(1-2\nu \right)-\rho^{*}/\rho^{s}}\;$$

#### 3.5.5. Establishment of Visco–Hyperelasticity–Air Constitutive Model

It is reported that the hyperelastic-viscoelastic-air behaviors are caused by the combination of hyperelasticity, viscoelasticity and air compressibility, which is $\sigma =\sigma^{e}+\sigma^{v}+\sigma^{p}$ [17,27], where $\sigma^{p}$= ΔP and $\sigma^{e}$=${\;\sigma }_{11}^{e}$. This assumption means that the stress–strain behaviors of PUME can be divided into quasi-static hyperelastic component ($\sigma^{e}$), the response related to rate and strain history ($\sigma^{v}$) and air compressibility ($\sigma^{p}$). We can obtain the finite strain hyperelastic–viscoelastic–air model of PUME.

(45) $$\begin{array}{cc} \sigma =-(p_{e}+p^{v})I & +\alpha_{1}B_{11}+\alpha_{2}B_{11}^{2}+F\left(t\right)\\ & \cdot \int_{0}^{t}\left\{B_{10}+B_{20}\left(I_{2}^{\prime }-3\right)+B_{30}{\left(I_{2}^{\prime }-3\right)}^{2}\right\},m\left(t-\tau \right)\dot{E}\left(\tau \right){d\tau F}^{T}\left(t\right)\\ & +\frac{P_{0}\varepsilon \left(1-2\nu \right)}{1-\varepsilon \left(1-2\nu \right)-\rho^{*}/\rho^{s}} \end{array}$$

Under uniaxial loading, if the stress is perpendicular to the loading direction, $\sigma_{22}$=$\sigma_{33}$=0 and $\sigma_{22}^{e}=\sigma_{33}^{e}$=0. We can then obtain the following equations from Equations (25) and (45).

(46) $$p^{v}=-\frac{1}{2}\;\cdot \lambda^{-1}\int_{0}^{t}\lambda^{-2}\;\left\{B_{10}+B_{20}\left(I_{2}^{\prime }-3\right)+B_{30}{\left(I_{2}^{\prime }-3\right)}^{2}\right\}.\exp\left(-\frac{t-\tau }{\theta_{i}}\right)\dot{E}\left(\tau \right)d\tau$$

(47) $$\sigma_{11}=2[(1-\varepsilon )-{\left(1-\varepsilon \right)}^{-2}]\;[\frac{\partial W}{\partial I_{1}}+{\left(1-\varepsilon \right)}^{-1}\frac{\partial W}{\partial I_{2}}]+{\left(1-\varepsilon \right)}^{2}\int_{0}^{t}\left(1-\varepsilon \right)\;\;\left\{B_{10}+B_{20}\left(I_{2}^{\prime }-3\right)+B_{30}{\left(I_{2}^{\prime }-3\right)}^{2}\right\}.\exp\left(-\frac{t-\tau }{\theta_{i}}\right)\dot{E}\left(\tau \right)d\tau +\frac{1}{2}\;\cdot {\left(1-\varepsilon \right)}^{-1}\;\overset{t}{\underset{0}{\int }}{\left(1-\varepsilon \right)}^{-2}\;\left\{B_{10}+B_{20}\left(I_{2}^{\prime }-3\right)+B_{30}{\left(I_{2}^{\prime }-3\right)}^{2}\right\}.\exp\left(-\frac{t-\tau }{\theta_{i}}\right)\dot{E}\left(\tau \right)d\tau +\;\frac{P_{0}\varepsilon \left(1-2\nu \right)}{1-\varepsilon \left(1-2\nu \right)-\rho^{*}/\rho^{s}}$$

#### 3.5.6. Application of Visco–Hyperelasticity–Air Constitutive Model

The compressive stress–strain data of PUME is the basis for its application in the design of energy-absorbing protective equipment and the simulation analysis of its application conditions in special fields such as collision and impact. Based on the microstructure characteristics of PUME and the stress–strain curves obtained from compression tests, an effective constitutive model is established, and the parameters of the constitutive model are obtained. In the future, when facing different collision conditions, it is necessary to select the stress–strain curve corresponding to the appropriate compression strain rate. The designers can input the corresponding strain rate into the constitutive model to obtain the stress–strain curve without further compression tests.

**Figure 16:**
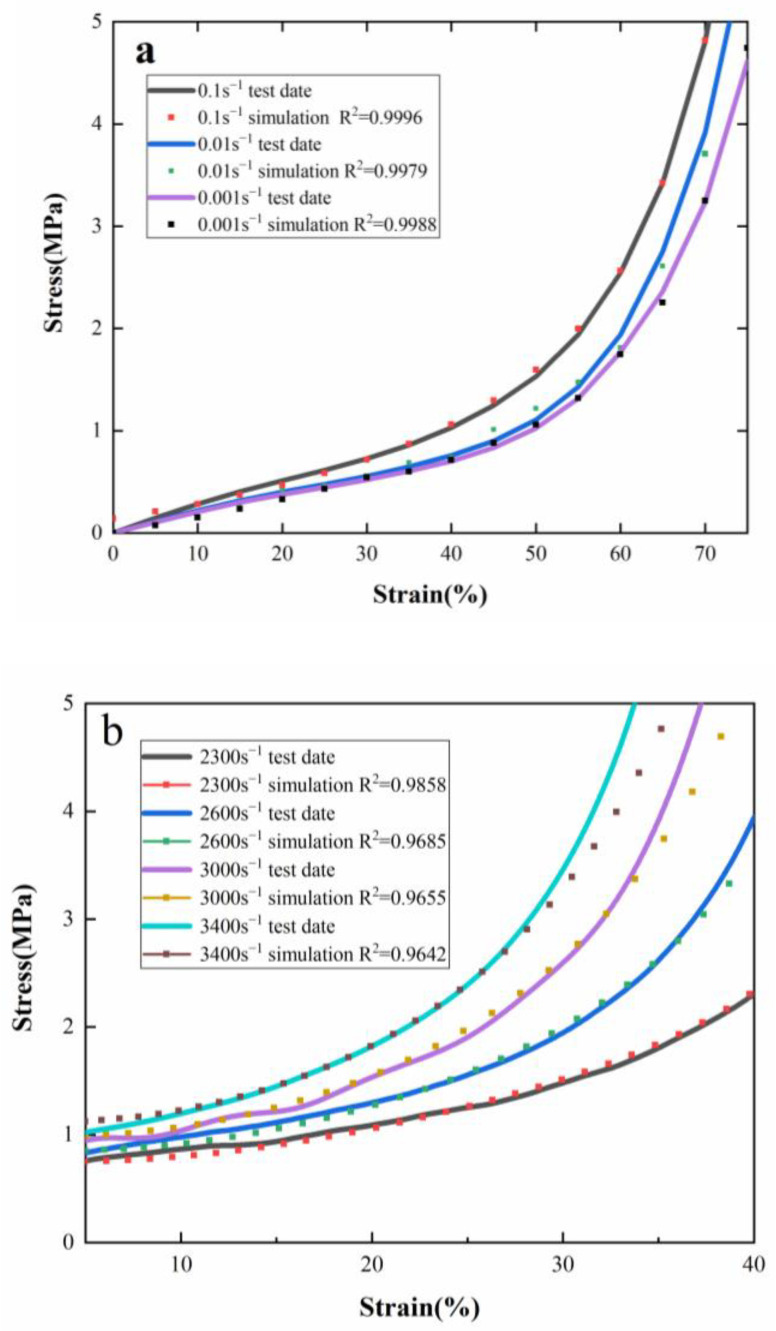
The fitting results between the experimental data and the model: (**a**) quasi-static test, (**b**) dynamic test.

The samples with a density of 600 kg/m^3^ at strain rates of 0.1 s^−1^ and 2300 s^−1^ were fitted by MATLAB software, and the constitutive model parameters and their lower and upper limits were obtained, which are listed in Table 5. With these determined parameters, the fitting points of 0.01 s^−1^, 0.001 s^−1^, 2600 s^−1^, 3000 s^−1^ and 3400 s^−1^ were obtained by the constitutive model. The least square method was used to compare the model with the experimental curve to obtain R-square. Figure 16 shows that the fitting points were in good agreement with the experimental test results, although the deviation of the high strain rate densification stage increased. The reason for this was that the parameters of the constitutive equation were determined by MATLAB software based on 2300 s^−1^, while the fitting points of 2600 s^−1^, 3000 s^−1^ and 3400 s^−1^ were directly obtained by the constitutive model. Equation (47) involved the product of $\dot{E}\left(\tau \right)$, and with the increasing of strain rate, the cumulative deviation of the densification stage increased. In the densification stage, the cells were completely compressed, which was almost equivalent to the performance of the material body. The model parameters can provide theoretical support for the simulation analysis and application of PUMEs in special fields such as collision, explosion, ballistic impact and other energy absorbing protective fields.

## 4. Conclusions

PUMEs have the properties of both foam and elastomer materials, so they can be used as an efficient energy absorbing material. In this study, PUMEs with different densities of 800 kg/m^3^, 600 kg/m^3^ and 400 kg/m^3^ were prepared. Infrared analysis showed that PUMEs formed a hard segment region and a soft segment region. Through the quasi-static mechanical tests and dynamic mechanical tests, it was found that the stress of PUME increased drastically with increasing strain rate and density. They are strain rate and density sensitive materials. The dynamic compression energy absorption and absorption efficiency diagrams of PUMEs with three kinds of density were illustrated, and the PUME with a density of 600 kg/m^3^ had the best energy absorption performance.

The visco–hyperelasticity–air constitutive model was established to describe the deformation response of PUME at high strain rates. The samples with 600 kg/m^3^ at different strain rates were fitted by MATLAB software, and the constitutive model parameters were obtained. The simulated results were in good agreement with the experimental results. In the future, simulation analysis and application research of PUMEs for energy absorbing protection could be further carried out by this model in special fields such as collision, explosion and ballistic impact.

## Figures and Tables

**Table 1:** The synthetic formula of PUMEs with different densities.

Sample kg/m^3^	MDI wt%	PTMEG1000 wt%	PTMEG2000 wt%	EP 330 wt%	BDO wt%	H_2_O wt%	AK7703 wt%	BDMAEE wt%	T-12 wt%
400	25	30	25.5	15	3.6	0.2	0.6	0.09	0.01
600	24.36	29.49	26.15	15.54	3.7	0.13	0.54	0.08	0.01
800	23.68	28.95	26.84	16.16	3.79	0.07	0.44	0.06	0.01

**Table 2:** Assignment of absorption bonds of FTIR spectra for PUME at 23 °C ± 2 °C.

Assignment	Wave Number (cm^−1^)
ν(O-H), C-OH stretching vibration	1099
δ (N-H) + ν(C-N), Amide III band	1223
ν(C-H), Hydrocarbon stretching vibration	1310
δ(N-H) + ν(C-N), Amide II band	1532
Ordered hydrogen-bonded urethane carbonyl	1705
Free urethane carbonyl	1729
ν(O-H), Hydroxyl stretching vibration	2858
Ν (C-H), -CH_2_-, Hydrocarbon stretching vibration	2934
Ν (N-H), Hydrogen-bonded N-H	3320

**Table 3:** Assignment of absorption bonds in carbonyl region of FTIR spectra for PUME.

Assignment	Wave Number (cm^−1^)	Ratio of Peak Area
Ordered hydrogen-bonded carbonyl	1690–1709	59.05
Disordered hydrogen-bonded carbonyl	1710–1724	0
Free carbonyl	1725–1740	40.95

**Table 4:** The meaning of model parameters [17].

Parameter	Nomenclature
A_i_	Hyperelastic material parameters
b_i_	Viscoelastic material parameters
B	Left Cauchy-Green deformation tensor
B_ij_	Components of B
C	Right Cauchy-Green deformation tensor
C_ij_	Components of C
E	Green strain tensor
E_ij_	Components of E
$\dot{e}$	Strain rate
F	Deformation gradient tensor
I_i_	Invariant of B (i = 1, 2, 3)
$I_{i}^{\prime }$	Invariant of C (i = 1, 2, 3)
t	Time
W	Strain energy potential
x	Position vector of matter particle at time t
X	Initial position vector of matter particles
λ	Extrude along the loading direction
$\dot{\lambda }$	Stretching rate
θ_i_	Relaxation time
σ	Cauchy stress tensor
σ_ij_	Components of σ
τ	Integral time variable
Ω	Constitutive functional
e	Elasticity
T	Tensor transpose
v	Viscoelastic
P_0_	Initial gas pressure (atmospheric pressure)
ν	Poisson’s ratio of material

**Table 5:** Parameters of constitutive model in compression test.

**Test**	$C_{01}$ **(MPa)**	$C_{10}$ **(MPa)**	$C_{20}$ **(MPa)**	$B_{10}$	$B_{20}$	$B_{30}$	$\;\;\theta_{i}$
quasi-static	0.08213 (0.0818, 0.08246)	−0.2949 (−0.2957, −0.2941)	−0.001653 (−0.001658, −0.001648)	0.9469 (0.3238, 1.57)	0.0554 (−0.2275, 0.3383)	−0.0007591 (−0.003309, 0.001791)	10
dynamic	0.4867 (0.4428, 0.5305)	−0.4063 (−0.4664, −0.3463)	0.4438 (0.4403, 0.4474)	0.0002236 (0.00004306, 0.0004042)	0.001505 (0.0006812, 0.002329)	0.9276 (0.5795, 1.276)	10^4^

## Data Availability

Not applicable.
